# Selecting Normalizers for MicroRNA RT-qPCR Expression Analysis in Murine Preimplantation Embryos and the Associated Conditioned Culture Media

**DOI:** 10.3390/jdb11020017

**Published:** 2023-04-04

**Authors:** David C. Hawke, Andrew J. Watson, Dean H. Betts

**Affiliations:** 1 Departments of Physiology and Pharmacology & Obstetrics and Gynaecology, Western University, London, ON N6A 3K7, Canada; 2 Children’s Health Research Institute—Lawson Health Research Institute, London, ON N6C 2R5, Canada

**Keywords:** preimplantation, embryo, microRNA, miRNA, murine, media, housekeeper, normalizer, reference, blastocyst

## Abstract

Normalizing RT-qPCR miRNA datasets that encompass numerous preimplantation embryo stages requires the identification of miRNAs that may be used as stable reference genes. A need has also arisen for the normalization of the accompanying conditioned culture media as extracellular miRNAs may serve as biomarkers of embryo developmental competence. Here, we evaluate the stability of six commonly used miRNA normalization candidates, as well as small nuclear U6, using five different means of evaluation (BestKeeper, NormFinder, geNorm, the comparative Delta Ct method and RefFinder comprehensive analysis) to assess their stability throughout murine preimplantation embryo development from the oocyte to the late blastocyst stages, both in whole embryos and the associated conditioned culture media. In descending order of effectiveness, miR-16, miR-191 and miR-106 were identified as the most stable individual reference miRNAs for developing whole CD1 murine preimplantation embryos, while miR-16, miR-106 and miR-103 were ideal for the conditioned culture media. Notably, the widely used U6 reference was among the least appropriate for normalizing both whole embryo and conditioned media miRNA datasets. Incorporating multiple reference miRNAs into the normalization basis via a geometric mean was deemed beneficial, and combinations of each set of stable miRNAs are further recommended, pending validation on a per experiment basis.

## 1. Introduction

MicroRNAs (miRNAs) are short, non-coding RNA sequences (~19–22 nt) that post-transcriptionally regulate cellular mRNA and, by extension, influence downstream pathway activation [1]. These sequences regulate many cellular processes owing to the nominal 8 bp mRNA binding requirement for interference with mRNA translation [2]. This widespread regulation has spurred their popularity, driven further by their potential use as diagnostic biomarkers owing to their considerable variety and extracellular stability [3]. miRNAs are expressed within a wide range of plants and animals, including mammalian preimplantation embryos [4,5] wherein miRNAs regulate developmentally critical features such as inner cell mass pluripotency and proliferation [6,7]. Extracellular miRNAs found within the surrounding spent culture media are also finding application as accessible biomarkers indicative of the developmental state [8]. Currently, the quantification of miRNA expression is most often performed using reverse transcription quantitative real-time polymerase chain reaction (RT-qPCR) technology, where mature transcripts are isolated, reverse-transcribed and quantified during subsequent amplification to determine the starting amount within the initial biological sample [9].

Reference genes, also referred to as normalizers, are used in relative RT-qPCR gene expression workflows and serve as stable reference points to accurately quantify true changes in target transcript abundance. Additionally, normalization to a reference gene enables the reliable and robust comparison of transcript abundance between datasets that were collected separately—either at a different time, in a different laboratory or by a different investigator—wherein slight deviations in either sample preparation (i.e., variable total RNA or sample quality) or analysis (i.e., variable detection thresholding) may have occurred. Normalizers should share biochemical properties with the targets to ensure consistent handling at the cellular level, during sample processing or during eventual analysis. Most often, authors normalize RT-qPCR data to a single reference gene; however, the geometric mean of multiple transcripts may be used to greater effect [10]. Several publicly available tools have been developed to assess panels of putative normalizers: (1) the comparative Delta Ct method [11], (2) geNorm [10], (3) BestKeeper [12], (4) NormFinder [13] and (5) RefFinder [14]. These methods evaluate expression data collected across a sample space of interest either by pairwise comparisons between candidate reference genes (1–3), using a mathematical model (4) or integrating and weighting these methods to provide a comprehensive analysis (5).

Gene expression studies utilizing whole preimplantation embryos must discern between changes in gene expression associated with normal development and those that are a physiological response to experimental perturbation. The complex nature of gene expression during normal preimplantation development invalidates the application of reference genes commonly accepted for many other cells and tissues, such as β-actin mRNA [15] or U6 snRNA [16]. In the context of miRNAs, RT-qPCR datasets comparing miRNA abundance in developing preimplantation embryos must contend with the occurrence of major transcriptional events such as zygotic gene activation, mid-preimplantation and cavitation-related transcriptional waves, and transcriptome remodeling resulting from active cell differentiation [17]. Between species, the timing of events such as zygotic gene activation can vary considerably [18]. Together, these events trigger the rapid degradation and expression of miRNAs to create a distinct expression profile during preimplantation development. Due to this considerable fluctuation, validated reference miRNAs are particularly important in order to capture the diverse miRNA character of this developmental period. Due to the lack of a consensus regarding preimplantation embryo miRNA normalizers, the individual normalizers used in studies investigating miRNA expression in preimplantation embryos and the surrounding culture media are diverse: U6 [19,20,21,22,23], miR-16-2 [24], miR-125b [25,26], 5S [27] and RPS18 [25]. To date, only a single study has validated preimplantation embryo miRNAs, and this is limited to the examination of bovine preimplantation embryos [16]. Additionally, no studies have validated stable extracellular miRNAs for normalizing early embryo conditioned media miRNA datasets; these datasets have diagnostic potential for the non-invasive selection of single human embryos for transfer during an in vitro fertilization cycle [8].

To address this, we have selected seven normalization candidates (six miRNAs and snRNA U6) based on their common use as reference genes within the miRNA literature. We have confirmed the expression of these candidates throughout murine preimplantation embryos and the associated conditioned media before evaluating their suitability for use as normalizers using the publicly available analytical tools. Based on this evaluation, we have ranked these individual candidates according to their effectiveness as normalizers for each of these datasets and evaluated the potential benefit of using the geometric mean of multiple candidates together in the normalization basis.

## 2. Materials and Methods

### 2.1. Animals and Preimplantation Embryo Collection

Animal use reporting follows the recommendations outlined in the ARRIVE guidelines [28]. CD1 female mice (Charles River Laboratories International Inc., Sherbrooke, QC, Canada) were housed in groups of 10 per cage under a 12 h day/night cycle (6 am to 6 pm) and allowed to feed ad libitum. Euthanasia was performed using CO_2_ asphyxiation followed by cervical dislocation, according to animal care protocol (AUP #2022-098) as approved by the Western University Animal Care Committee. Two batches of 20 three- to four-week-old female CD1 mice were intraperitoneally injected with 5 IU of pregnant mare’s gonadotropin (PMSG; Intervet Canada Ltd., Whitby, ON, Canada) followed by a second injection of 5 IU of human chorionic gonadotropin (hCG; Intervet Canada Ltd., Whitby, ON, Canada) to induce superovulation. Following the second injection, females were immediately housed with a male of at least 8 weeks of age at a ratio of 2:1 overnight. For zygote collection, plugged female mice were sacrificed 15 h after hCG injection and cumulus oocyte complexes were collected from the oviducts in warmed M2 medium before being treated briefly with M2 media containing hyaluronidase (Sigma-Aldrich, P/N: MR-051-F) at 37 °C. Ovulated MII oocytes were similarly collected from abstinent females. For 2-cell embryos, plugged female mice were sacrificed 48 h after hCG injection and 2-cell embryos were flushed from the oviducts in warmed M2 Medium (Sigma Aldrich, Oakville, ON, Canada; P/N: MR-015-D).

### 2.2. Embryo Culture for Developmental Series Sample Preparation

The 2-cell embryos were cultured at 20 per 20 µL microdroplet of KSOMaa medium supplemented with BSA (EmbryoMax; Sigma Aldrich, P/N: MR-106-D) under a mineral oil blanket at 37 °C in a 5% O_2_, 5% CO_2_ nitrogen atmosphere. At various morphological stages (2 cells, 4–8 cells, morula, blastocyst, and expanded blastocyst), 10 embryos were collected and immediately lysed in 2 µL of Cells-to-Ct ‘Lysis Solution’ (Thermo Fisher Scientific, Mississauga, ON, Canada; P/N: 4391848) before being stored at −20 °C. Ovulated MII oocytes and zygotes were similarly lysed and stored following hyaluronidase treatment. Upon thawing, 10 µL of ‘Lysis Solution’ containing a 1:100 dilution of DNase I (Thermo Fisher Scientific; P/N: 18047019) was added to each sample followed by a 2 µL addition of ‘Stop Solution’ (Thermo Fisher Scientific; P/N: 4402960) 8 min later. Samples were vortexed and stored at −20 °C before subsequent reverse transcription. A total of 210 embryos were collected for the entire dataset.

### 2.3. Embryo Culture for Media Conditioning Sample Preparation

The 2-cell embryos were flushed and cultured in groups of 20 in KSOMaa with 0.1% PVA (no BSA), made fresh in-house. A fresh blank microdroplet was cultured during this period in tandem to serve as a background negative control. Every 12 h, the embryos were washed and moved to fresh new microdroplets. After embryo removal, 15 µL of the spent and control microdroplets were then collected separately and each was immediately lysed with an addition of 15 µL ‘Lysis Solution’ containing 1% DNase I, followed by a 3 µL addition of ‘Stop Solution’ 8 min later. Samples were stored at −20 °C for later analysis using droplet digital PCR. Conditioned media samples were generated using a total of 60 embryos that were moved to new microdroplets every 12 h.

### 2.4. Reverse Transcription Quantitative Real-Time PCR (RT-qPCR)

Reverse transcription of whole embryo lysate samples was performed using a MicroRNA Reverse Transcription kit (Thermo Fisher Scientific, P/N: 4366596) in addition to separately ordered TaqMan miRNA primers (Thermo Fisher Scientific, P/N: 4427975) for each of the 6 targeted miRNAs and snU6 according to the manufacturer’s instructions: 10 µL of RT Mastermix, 1 µL of whole embryo lysate and 4 µL of water were added to each RT reaction before thermal cycling at 16 °C for 30 min, 42 °C for 30 min and 85 °C for 5 min in both cases. For qPCR amplification of whole embryo lysate samples, 1.33 µL of RT reaction products was mixed with 18.67 µL of TaqMan 2X Universal PCR Mastermix No AmpErase UNG (Thermo Fisher Scientific, P/N: 4324018), and quantitative amplification was performed using a CFX384 thermocycler (BioRad, Mississauga, ON, Canada) for 40 cycles.

### 2.5. Reverse Transcription Droplet Digital PCR (RT-ddPCR) 

Reverse transcription of the conditioned media samples was performed similarly using a TaqMan MicroRNA Reverse Transcription kit (Thermo Fisher Scientific, P/N: 4366596) and TaqMan RT primers (Thermo Fisher Scientific, P/N: 4427975) for the seven normalization candidates. Conditioned media samples were thawed, and 2.5 µL aliquots of each were mixed with 7 µL of TaqMan RT Mastermix, 2.5 µL of MilliQ sterilized H_2_O and 3 µL of TaqMan RT primer for each target candidate sequence to yield seven separate reactions for each media sample. For amplification, 7 µL of RT products was mixed with 10 µL of ddPCR Supermix for probes (BioRad Laboratories, Mississauga, ON, Canada P/N: 1863010) and 1 µL of their respective TaqMan probes (Thermo Fisher Scientific, P/N: 4427975). Amplification was performed using a BioRad thermal cycler according to the manufacturer’s instructions (25 °C, 3 min; 95 °C, 10 min; 40× (94 °C, 30 s; 60 °C, 1 min); 98 °C for 10 min). The total volume containing amplification products was mixed with 20 µL droplet generation oil (BioRad Laboratories, P/N: 1863005) using a BioRad QX200 Droplet Generator (BioRad Technologies, P/N: 1864002) according to manufacturer’s guidelines, followed by quantification using a BioRad QX200 Droplet Digital PCR System (BioRad Technologies, P/N: 1864001). Three biological replicates and two technical replicates of each were performed for each pair of RT and PCR reactions, and the average was taken. In several instances, either a single technical replicate was used in absence of a successful second repetition or two biological replicates were averaged instead of three.

### 2.6. Data Analysis

The following publicly available methods and tools were used for reference gene evaluation: the comparative Delta Ct method [11], geNorm v.38 (Microsoft Excel software package) [10], BestKeeper Version 1 (Microsoft Excel spreadsheet template) [12], NormFinder 0.953 (Microsoft Excel Add-In) [13] and the integrated RefFinder approach (https://www.heartcure.com.au/reffinder; accessed on 2 February 2022) [14]. For the comparative Delta Ct method, whole lysate RT-qPCR data were analyzed in their native base 2 logarithmic form (raw Ct values) and conditioned media RT-ddPCR copy number data were base 2-logarithmically transformed before analysis. For geNorm analysis, both cycle threshold RT-qPCR and RT-ddPCR datasets were converted to relative expression values before input into the geNorm Excel software package. For BestKeeper analysis, whole lysate RT-qPCR data were analyzed as is (raw Ct values) and base 2 logarithmic transformations were performed on the conditioned media copy number data prior to analysis. RT-qPCR and RT-ddPCR datasets were converted to relative values before input into the NormFinder Excel Add-In. All RefFinder input data for both RT-qPCR and RT-ddPCR sample sets were base 2-logarithmically transformed before analysis.

## 3. Results

### 3.1. Candidate Stability Analysis

Six miRNA candidates commonly used as normalizers for human and murine miRNA datasets (Schwarzenbach et al. [29]) that were also expressed at significant levels across murine preimplantation development were selected [30,31,32]: let-7a [33,34,35], miR-16 [34,35,36,37,38,39], miR-26a [33,40], miR-103 [33,41], miR-106 [35,39,41] and miR-191 [38,39,42,43,44] (Table 1). Additionally, a frequently used non-miRNA normalizer for miRNA datasets, snRNA U6 [38], was also chosen.

**Figure 1:**
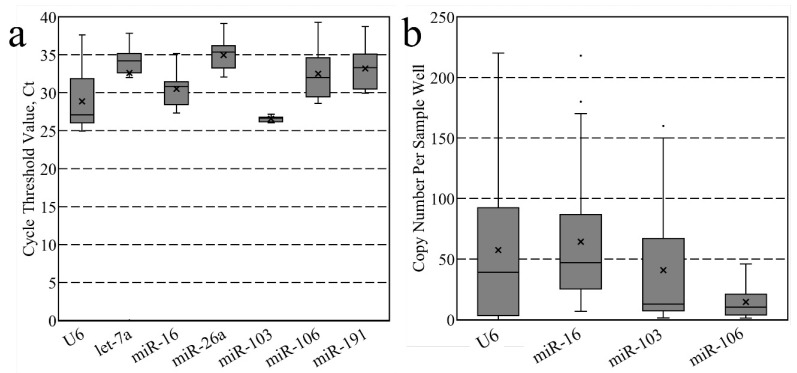
Candidate abundance across murine preimplantation development for both whole embryos and conditioned media sample sets. (**a**) Raw cycle threshold values, Ct, from RT-qPCR analysis for each candidate during murine preimplantation development (ovulated MII oocyte, zygote, two-cell, four-cell, morula, blastocyst, and expanded blastocyst) and (**b**) candidate copy number values per sample well from RT-ddPCR analysis of microdroplets conditioned in 12 h periods with embryos from the two-cell stage to the expanded blastocyst. Each box represents the inner two quartiles (Q1–Q3) of each candidate miRNA Ct data subset, the x markers in each box indicate the mean Ct value or copy number for each miRNA candidate across the sample set, the box inner lines represent each sample set’s median values, the whiskers represent the maxima and minima and the dots beyond the whiskers represent outliers. For whole embryo samples in (**a**), each average represents the average of 21 samples derived from 210 whole embryos, and for media samples (**b**), each average represents the average of 18 samples taken across development using 60 embryos to continually condition fresh media microdroplets during for each 12 h period.

For both whole murine preimplantation embryo and associated conditioned media datasets, these candidates were assayed using either RT-qPCR or RT-ddPCR workflows, respectively (Figure 1). The candidates were then evaluated as possible normalizers using five different methods: (1) the comparative Delta Ct method [11], (2) geNorm [10], (3) BestKeeper [12], (4) NormFinder [13] and (5) RefFinder [14]. The tabulated rankings of candidate stability as determined by each method are listed in Table 2 for the whole lysate samples and in Table 3 for the conditioned media samples.

**Figure 2:**
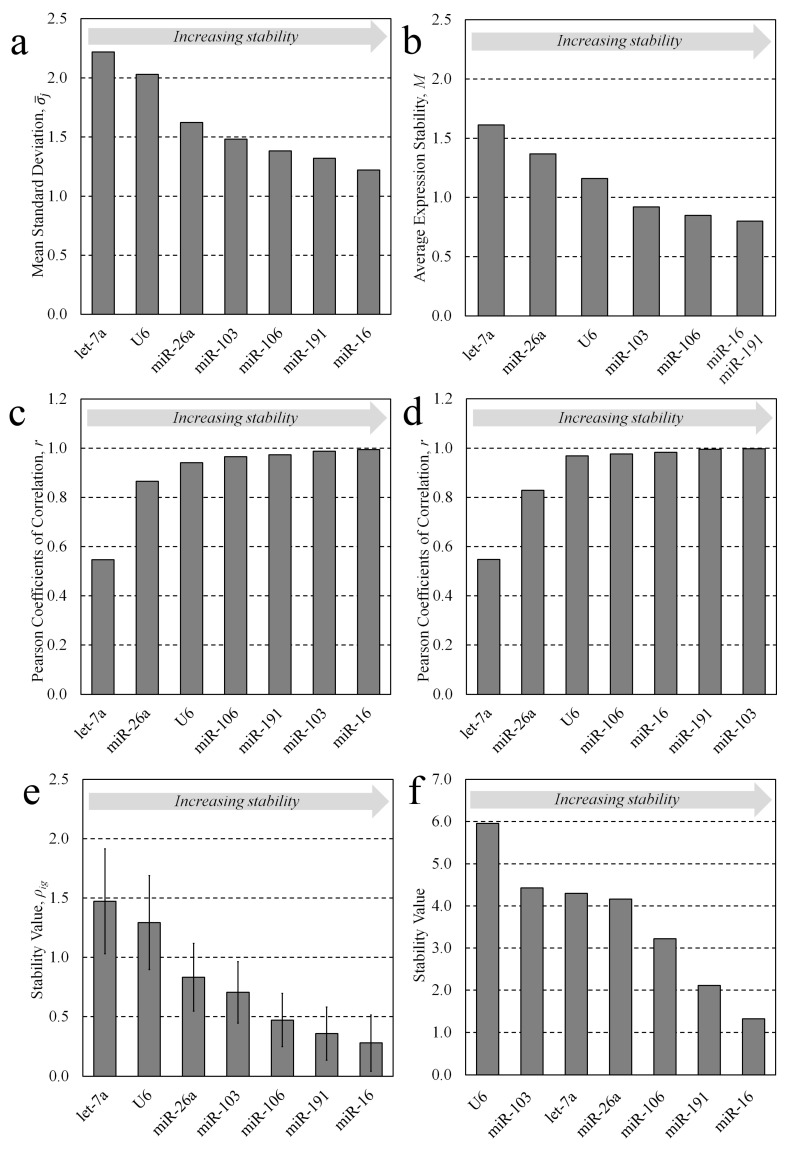
Reference candidate stability metrics for staged murine whole embryos (ovulated MII oocytes, zygotes, 2-cells, 4-cells, morulae, blastocysts, and expanded blastocysts). (**a**) The average standard deviation of each reference candidate’s pairwise comparisons calculated using the comparative Delta Ct method. (**b**) The average expression stability factor, M, of pools of reference candidates as calculated using the geNorm method. (**c**) The Pearson coefficients of correlation calculated using the BestKeeper method. (**d**) The Pearson coefficients of correlation according to the repeated BestKeeper method. (**e**) The stability values calculated for each candidate using the NormFinder method; errors bars indicate the standard error of the mean. (**f**) The integrated stability values of each reference candidate calculated using the comprehensive RefFinder method.

**Figure 3:**
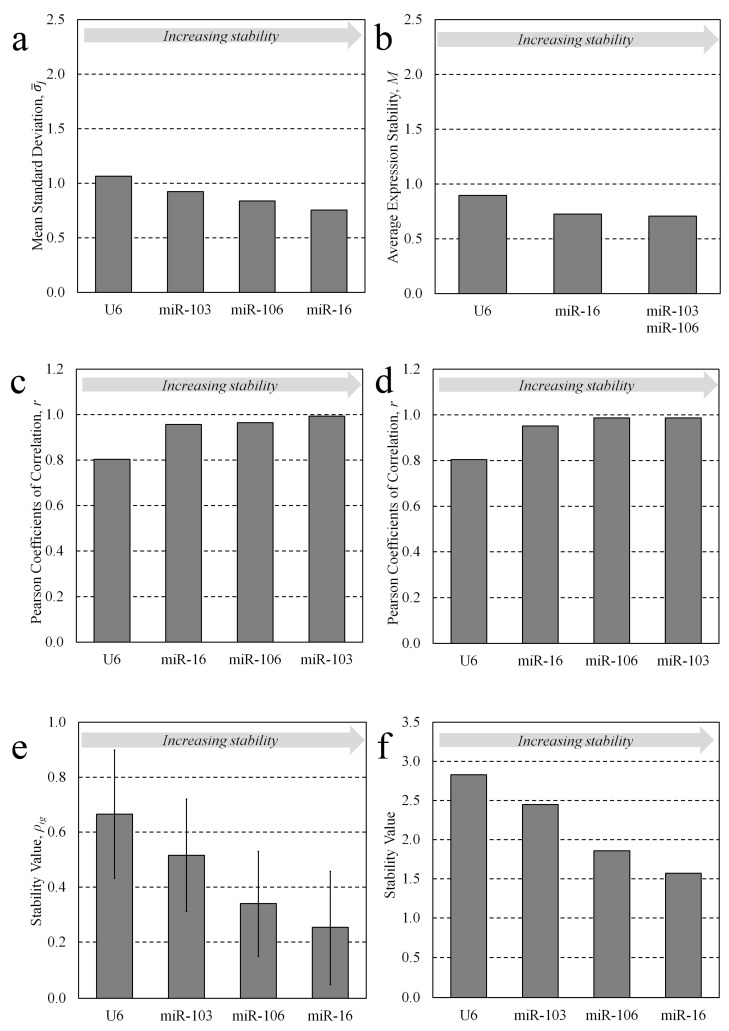
Reference candidate stability metrics for media samples conditioned with developing murine preimplantation embryos (12 h conditioning periods from the 2-cell stage to the expanded blastocyst stage embryos, collected continuously). (**a**) The average standard deviation of each reference candidate’s pairwise comparisons calculated using the comparative Delta Ct method. (**b**) The average expression stability factor, M, of pools of reference candidates according to the geNorm method. (**c**) The Pearson coefficients of correlation as calculated using the BestKeeper method. (**d**) The Pearson coefficients of correlation calculated using the iterative BestKeeper method. (**e**) The stability values for each candidate according to the NormFinder method; errors bars indicate the standard error of the mean. (**f**) The stability values for expression of each reference candidate using the integrated RefFinder method.

The comparative Delta Ct method [11] utilizes a simple pairwise comparison approach and determines the variability in the expression differences between all possible pairs of candidate expression values in each individual sample group. Using this method, the average standard deviation, ${\overset{-}{\sigma }}_{j}$, of the expression differences between each of the seven normalization candidates in whole preimplantation embryo developmental stages (oocyte, zygote, 2-cell, 4-cell, morula, blastocyst, and expanded blastocyst) and each of the four candidates for conditioned media (2-cell embryo to expanded blastocyst; 12 h periods) were calculated. The candidates were, respectively, ranked from the lowest standard deviation to the greatest (most stable to the least stable) and the average standard deviation of each candidate across sample groups is listed in parentheses. For whole embryos: 1. miR-16 (1.220), 2. miR-191 (1.321), 3. miR-106 (1.383), 4. miR-103 (1.482), 5. miR-26a (1.623), 6. snRNA U6 (2.029), 7. let-7a (2.218) (Figure 2a). For conditioned media: 1. miR-16 (0.755), 2. miR-106 (0.839), 3. miR-103 (0.925), 4. snRNA U6 (1.066) (Figure 3a).

Using the geNorm method [10], the candidates were ranked according to their calculated stability value across preimplantation embryo developmental stages for both sample sets. Like the Delta Ct method, the geNorm method makes all possible candidate pairwise comparisons at each developmental stage and determines the average interstage standard deviation for each candidate. The standard deviations of all candidates are averaged and reported as an average expression stability value, M. The least stable candidate is removed from the pool and M is recalculated, and this process continues until only two candidates remain. A lower expression stability value is indicative of greater sequence stability and better utility as a normalization sequence; however, any candidate yielding an individual stability value, M_j_, below the consensus cut-off of 1.5 was considered adequate. The candidates were ranked as follows, from greatest stability to least, with the calculated stability value M_j_ listed in parentheses for each. For whole embryo lysates: 1. miR-16/miR-191 (0.799), 3. miR-106 (0.923), 4. miR-103 (0.991), 5. snRNA U6 (1.522), 6. miR-26a (1.783), 7. let-7a (2.218) (Figure 2c). For conditioned media: 1. miR-103/miR-106 (0.707), 3. miR-16 (0.736), 4. snRNA U6 (1.066) (Figure 3c). Among the whole lysate sample groups, miR-16, miR-191, miR-106 and miR-103 were below the cut-off, along with all media candidates—miR-103, miR-106, miR-16 and U6—indicating that these are acceptable normalizers.

The BestKeeper method [12] was used to assess the pairwise correlation of each candidate across these stages for both whole lysate and conditioned media samples. The BestKeeper approach uses a pairwise correlation strategy that effectively ranks candidates according to their degree of correlation with an index that is calculated as the geometric mean of all candidates within a comparison pool. To numerically assess the extent of correlation with the index, the Pearson coefficients of correlation, r, are calculated; candidates with an r value closer to 1 were more correlative and deemed more stable. The panel of candidates were ranked for their appropriateness as normalizers accordingly, from most to least stable, with their coefficients listed in parentheses. For whole embryo lysates: 1. miR-16 (0.994), 2. miR-103 (0.987), 3. miR-191 (0.973), 4. miR-106 (0.965), 5. snRNA U6 (0.941), 6. miR-26a (0.865), 7. let-7a (0.547) (Figure 2b). For conditioned media: 1. miR-103 (0.993), 2. miR-106 (0.965), 3. miR-16 (0.957), 4. snRNA U6 (0.804) (Figure 3b).

To discount sensitivity to poorly performing candidates included within the initial selection pool, a second ‘repeated’ BestKeeper analysis was also performed. In the repeated analysis, the Pearson correlations were calculated; however, the least-conforming candidate was then ‘dropped’ from the pool and the index was recalculated, along with the coefficients for the remaining candidates. This was repeated until only two candidates remained, and the rankings are reported according to the ordering of ‘drop out’, with the first candidate to be removed receiving the lowest rank; each candidate’s Pearson correlation coefficient during the iteration of exclusion is listed in parentheses. For whole embryo lysates: 1. miR-103 (0.997), 2. miR-191 (0.995), 3. miR-16 (0.983), 4. miR-106 (0.976), 5. snRNA U6 (0.968), 6. miR-26a (0.829), 7. let-7a (0.547). For conditioned media: 1. miR-103/miR-106 (0.987), 3. miR-16 (0.951), 4. snRNA U6 (0.804) (Figure 3b). The BestKeeper outputs including crossing point data, Pearson correlation coefficients and regression analysis are listed in Tables S1–S3, respectively (see Supplementary Materials). All datasets were logarithmically transformed (base 2) before analysis.

The NormFinder method [13] evaluates and ranks reference candidates using a model-based approach that calculates a stability value, $\rho_{ig}$, that is a descriptor of both inter- and intra-group variability. Similar to the Delta Ct and geNorm methods, a lower stability value reflects greater sequence stability and utility as a reference gene for data normalization. By this method, the candidates were ranked accordingly, from most stable to least, with each associated stability value in parentheses. For whole embryos: 1. miR-16 (0.277), 2. miR-191 (0.356), 3. miR-106 (0.471), 4. miR-103 (0.704), 5. miR-26a (0.832), 6. snRNA U6 (1.293), 7. let-7a (1.472) (Figure 2d). For conditioned media: 1. miR-16, 2. miR-106, 3. miR-103, 4. snRNA U6 (Figure 3d).

The comprehensive ranking, a weighted ranking of each previous method, was determined using the integrated approach RefFinder [14]. A composite stability value based on a weighted integration of these methods was calculated for the candidates for both sample sets. For whole embryos: 1. miR-16 (0.277), 2. miR-191 (0.356), 3. miR-106 (0.471), 4. miR-103 (0.704), 5. miR-26a (0.832), 6. snRNA U6 (1.293), 7. let-7a (1.472) (Figure 2e). For conditioned media: 1. miR-16 (0.253), 2. miR-106 (0.340), 3. miR-103 (0.515), 4. snRNA U6 (0.665) (Figure 3e).

### 3.2. Multiple Candidate Stability Analysis

**Figure 4:**
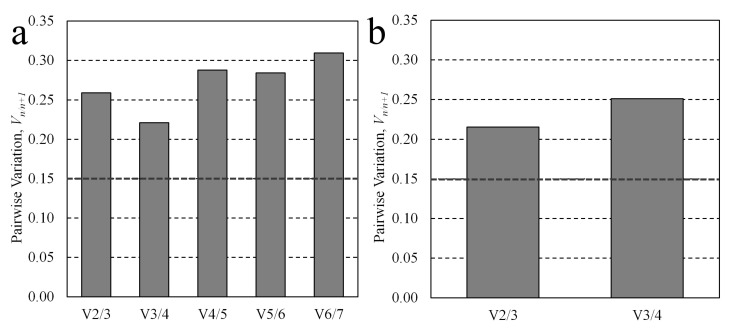
Pairwise variation, V_n/n+1_, of the normalization basis assessing the addition of the next most stable reference candidate. The benefit of including additional reference genes in the calculation of the normalization factor was examined as a means of assessing the impact of a new addition to the normalization factor, according to Vandesompele [10], for (**a**) whole oocytes and embryos, and (**b**) conditioned media samples. A pairwise variation value of V_n/n+1_ > 0.15 (represented by the dashed horizontal line) indicates that the addition of another candidate to the basis had a substantial impact on the intergroup geomean variability, indicating that further improvement to the basis may be achieved by inclusion of the n + 1 candidate.

To determine if including multiple reference candidates within the normalization basis would be beneficial to the reference stability, the pairwise variation, V_n/n+1_, was calculated for subsets of each sample set’s candidate pool using a feature of the geNorm tool [10]. The pairwise variation, V_n/n+1_, describes the relative degree of inter-group variation that occurs between normalization bases—calculated as the geometric mean of the candidates, the normalization factor—that occurs when the next most stable candidate is added to the basis. As proposed by Vandesompele, values above a cut-off pairwise variation threshold (V_n/n+1_ > 0.15) were considered indicative that the addition of the next normalization candidate to the basis expansion was impactful. For whole embryo lysates, the incorporation of additional candidates into the geometric mean continued to significantly influence the geomean as pairwise variation was greater than 0.15 for every addition. Similarly, for conditioned media samples, significant pairwise variation was observed for the expansion of the miR-103/miR-106 normalization factor to also include both miR-16 and snRNA U6, suggesting that the addition of both may be beneficial. The pairwise variation values are plotted in Figure 4a,b.

## 4. Discussion

Normalization to stable endogenous miRNA references is essential for accurate quantification of miRNA expression using RT-qPCR. It is particularly important for preimplantation embryo RT-qPCR datasets as it avoids the technical uncertainty associated with handling such limited amounts of total RNA. Furthermore, the sharp rise in an embryo’s total RNA content after the 8-cell stage [45,46] renders the use of alternative normalization bases, such as embryo count and exogenous spike-ins, inadequate. These issues are further exacerbated when analyzing the spent culture media.

Every candidate evaluation method ranked miR-16, miR-191, miR-106 and miR-103 above miR-26a, snRNA U6 and let-7a across the 210 whole embryos comprising the lysate samples and, similarly, miR-16, miR-106 and miR-103 over snRNA U6 in the media samples conditioned continuously with 60 embryos. Furthermore, let-7a, miR-26a and snRNA U6 failed to satisfy an internal geNorm metric (M_j_ = 1.5) assessing the average standard deviation of their pairwise comparisons, indicating that they are inappropriate as normalizers. These similar results between sample sets were expected, since, for short conditioning times (<24 h), we have previously shown the extraembryonic ‘miRnome’ composition to be reflective of the whole embryos [47]. The comparison of the two ‘drop-out’ methods included in our analysis, geNorm and repeated BestKeeper, to their respective non-iterative counterparts, the comparative Ct method and BestKeeper, indicated the minimal impact of poorly performing candidates on the overall evaluation. Since effective reference candidates must only share a similar expression profile with their targets, we postulated that these candidates may be applicable in other species, despite differences in species-specific timing of RNA expression due to zygotic gene activation. To see if any of these reference candidates could be accepted as an interspecies preimplantation standard, we compared these results to other published preimplantation reference miRNA data. Our results were consistent with the previously reported stability of miR-191 and miR-103 and inadequacy of snRNA U6, let-7a and miR-26a in bovine preimplantation embryos [16], in addition to the reported stability of miR-191 in rabbit preimplantation embryos [48]. This interspecies agreement along with the high degree of miRNA sequence conservation may suggest that miR-191, in particular, is appropriate for other model organisms, in addition to human preimplantation embryos, for which normalization data may be scarce or unavailable.

We noticed that our most stable candidates (miR-16, miR-191, miR-106, miR-103) were both derived from oocytic reserves and expressed during early development, while the least stable were either nearly exclusively oocyte-derived (let-7a and miR-26a) or exhibited profound maternal-to-embryonic transition (MET) degradation followed by sharp embryonic expression (snRNA U6) [19]. The poor suitability of snRNA U6 is notable considering its frequent use as a normalizer in miRNA preimplantation embryo studies [20,21,22,23].

The inclusion of multiple miRNAs in the normalization basis may improve its stability [10]; indeed, the impact of utilizing different individual normalizers to draw conclusions from a single dataset, even among those deemed stable, is substantial [16,35]. The significant pairwise variation in whole lysate geomeans resulting from the continual addition of the next stable normalizer is consistent with other preimplantation embryo studies [15,16] and indicates that each addition to the basis was significantly impacting the interpretation of the dataset. V_n/n+1_ did not drop below the value of 0.15, indicating that the addition of the top-ranking miRNA normalizers to the basis impacted the normalization significantly. This is due to the diverse expression profiles of the candidates (i.e., maternal vs. embryonic miRNA origins, non-miRNA snU6), whereby even the top-ranking miRNAs exhibited significantly different expression profiles from one another. To better improve the normalization basis using a geometric mean, a larger panel of miRNA candidates should be evaluated. Based on these results, we recommend a combination of either miR-16/miR-191 or miR-16/miR-191/miR-106 for whole embryo lysates and a combination of miR-16/miR-191 for the conditioned media.

We performed a literature search to investigate whether these miRNAs remained inert with regard to several commonly studied oocyte factors such as oocyte age and maturity. As part of clinical assisted reproduction, human preimplantation embryos are frequently derived from oocytes originating from donors of advanced maternal age, and oocytes retrieved during stimulated cycles are of varying degrees of maturity [49], for which maturation may be performed in vitro prior to intracytoplasmic sperm injection (ICSI) fertilization [50]. Our assessment of the literature did not reveal any differential expression of the candidate miRNAs during in vitro bovine oocyte maturation [19,27] except for a single instance of miR-106 displaying a 2.87-fold increase between the MII and GV stages [51]. For aged oocytes, the expression of let-7a, miR-16, miR-26a, miR-103 and miR-191 was consistent between GV oocytes from aged (14–16-month-old) vs. young (4–6-week-old) mice [52], and none of the identified candidate miRNAs (miR-16, miR-26a, miR-103, miR-106, miR-191) varied significantly in abundance between human oocytes from younger and older patients [53]. Additionally, human inter-oocyte expression of miR-16, miR-106 and miR-191 was variable, which has also been reported for miR-26a and miR-103 [53].

Our study is limited by the size of our candidate panels, restricting our recommendations to the handful of miRNAs that we selected. In particular, our media panel only contained four highly abundant, detectable candidates from the whole lysate panel, and expansion to include more candidates with abundance in these samples would benefit these analyses. Larger panels are necessary to identify more stable miRNA normalizers as well as enable the accurate establishment of a multi-miRNA geometric mean basis. We also note that while choosing normalization miRNAs that are central to core cellular processes is an effective strategy to identify robust normalizers, it also predisposes these candidates to be ubiquitously expressed across many other tissues and cell types, including other species. This means that preimplantation embryo culture media, which are routinely formulated with miRNA-containing serum albumin, could potentially contain a pre-existing population of these miRNAs, and this should be controlled for on a case-by-case basis [54,55]. Finally, the miRNA normalizers proposed in this study should be further evaluated on a per-experiment basis to ensure they remain inert to experimental perturbations.

## Figures and Tables

**Table 1:** Reference candidates selected for stability evaluation.

Candidate Name	NCBI/miRBase Accession Number	TaqMan Assay ID	Reference(s)
U6	NR_004394	001973	[20,21,22,23,37]
let-7a-5p	MIMAT0000521	000377	[32,33,34]
miR-16-5p	MIMAT0000527	000391	[33,34,35,36,37,38]
miR-26a-5p	MIMAT0000533	000405	[32,39]
miR-103-3p	MIMAT0000546	000439	[32,40]
miR-106a-5p	MIMAT0000385	002459	[33,38,40]
miR-191-5p	MIMAT0000221	002299	[37,38,41,42,43]

**Table 2:** Summary of reference candidate rankings according to each method of evaluation for whole embryo lysates.

Ranking Order							
Method	1	2	3	4	5	6	7
Delta CT	miR-16	miR-191	miR-106	miR-103	miR-26a	U6	let-7a
geNorm	miR-16|miR-191		miR-106	miR-103	U6	miR-26a	let-7a
BestKeeper	miR-16	miR-103	miR-191	miR-106	U6	miR-26a	let-7a
BestKeeper (repeated)	miR-103	miR-191	miR-16	miR-106	U6	miR-26a	let-7a
Normfinder	miR-16	miR-191	miR-106	miR-103	miR-26a	U6	let-7a
RefFinder	miR-16	miR-191	miR-106	miR-26a	let-7a	miR-103	U6

**Table 3:** Summary of reference candidate rankings according to each method of evaluation for conditioned media samples.

Ranking Order				
Method	1	2	3	4
Delta CT	miR-16	miR-106	miR-103	U6
geNorm	miR-103|miR-106		miR-16	U6
BestKeeper	miR-103	miR-106	miR-16	U6
BestKeeper (repeated)	miR 103|miR-106		miR-16	U6
Normfinder	miR-16	miR-106	miR-103	U6
RefFinder	miR-16	miR-106	miR-103	U6

## Data Availability

The data presented in this study are openly available in a Mendeley Data repository at https://data.mendeley.com/datasets/3t65chv233/1, accessed on 18 January 2023.

## Supplementary Materials

The following supporting information can be downloaded at: https://www.mdpi.com/article/10.3390/jdb11020017/s1, Table S1: Dataset characteristics of whole embryo lysate sample set data and conditioned media samples according to the BestKeeper analytical tool; Table S2: Pearson correlation coefficients describing inter-candidate correlation and correlation with the BestKeeper index, according to the BestKeeper tool for whole embryo lysates and conditioned media sample sets; Table S3: Regression output describing individual candidate correlation with the BestKeeper index according to the BestKeeper tool for whole embryo lysates and conditioned media sample sets.
