# Supplementary material for: Selecting Normalizers for MicroRNA RT-qPCR Expression Analysis in Murine Preimplantation Embryos and the Associated Conditioned Culture Media
Source: J Dev Biol. 2023 Apr 4;11(2):17. doi: 10.3390/jdb11020017 (PMC10123758; doi:10.3390/jdb11020017)
Supplement: Supplementary file 1 [file jdb-11-00017-s001.zip › Table S1.pdf]

Table S1: Dataset characteristics of whole embryo lysate sample set data (raw Ct values) and conditioned media samples (base 2 logarithmic transformations) according to the BestKeeper analytical tool. *Abbr. CP – crossing point (cycle threshold)*

CP data of housekeeping Genes (Whole Embryo Lysates):

	U6	let-7a	miR-16	miR-26a	miR-103	miR-106	miR-191
**n**	7	7	7	7	7	7	7
**geo Mean [CP]**	28.58	34.37	30.50	34.92	32.36	33.01	33.45
**ar Mean [CP]**	28.76	34.39	30.56	34.96	32.49	33.10	33.53
**min [CP]**	25.39	32.20	27.41	32.22	28.69	30.05	30.30
**max [CP]**	35.01	35.92	33.45	36.61	36.58	37.26	36.47
**std dev [± CP]**	2.89	1.08	1.64	1.43	2.58	2.07	2.17
**CV [% CP]**	10.04	3.14	5.37	4.10	7.94	6.26	6.46
**min [x-fold]**	-9.12	-4.50	-8.54	-6.49	-12.74	-7.78	-8.85
**max [x-fold]**	86.32	2.93	7.72	3.21	18.64	18.99	8.11
**std dev [± x-fold]**	7.40	2.11	3.12	2.70	5.97	4.21	4.49

CP data of housekeeping Genes (Conditioned Media):

	U6	miR-16	miR-103	miR-106
**n**	6	6	6	6
**geo Mean [CP]**	5.50	5.31	3.92	2.91
**ar Mean [CP]**	5.57	5.44	4.24	3.16
**min [CP]**	4.05	4.06	2.62	1.96
**max [CP]**	6.41	7.50	7.17	5.07
**std dev [± CP]**	0.75	1.05	1.60	1.22
**CV [% CP]**	13.55	19.22	37.67	38.53
**min [x-fold]**	-2.73	-2.38	-2.47	-1.93
**max [x-fold]**	1.88	4.57	9.56	4.47
**std dev [± x-fold]**	1.69	2.06	3.02	2.33
