# Supplementary material for: Selecting Normalizers for MicroRNA RT-qPCR Expression Analysis in Murine Preimplantation Embryos and the Associated Conditioned Culture Media
Source: J Dev Biol. 2023 Apr 4;11(2):17. doi: 10.3390/jdb11020017 (PMC10123758; doi:10.3390/jdb11020017)
Supplement: Supplementary file 1 [file jdb-11-00017-s001.zip › Table S2.pdf]

Table S2: Pearson correlation coefficients describing inter-candidate correlation and correlation with the BestKeeper index, according to the BestKeeper tool for whole embryo lysates and conditioned media sample sets.

Pearson correlation coefficient, r - Whole Embryo Lysates

vs.	U6	let-7a	miR-16	miR-26a	miR-103	miR-106	miR-191
**let-7a**	0.287	-	-	-	-	-	-
**p-value**	0.533	-	-	-	-	-	-
**miR-16**	0.909	0.608	-	-	-	-	-
**p-value**	0.005	0.148	-	-	-	-	-
**miR-26a**	0.681	0.885	0.891	-	-	-	-
**p-value**	0.092	0.008	0.007	-	-	-	-
**miR-103**	0.952	0.443	0.967	0.798	-	-	-
**p-value**	0.001	0.321	0.001	0.032	-	-	-
**miR-106**	0.940	0.397	0.960	0.754	0.951	-	-
**p-value**	0.002	0.377	0.001	0.050	0.001	-	-
**miR-191**	0.894	0.478	0.961	0.809	0.985	0.938	-
**p-value**	0.007	0.277	0.001	0.027	0.001	0.002	-

BestKeeper vs.	U6	let-7a	miR-16	miR-26a	miR-103	miR-106	miR-191
**coeff. of corr. [r]**	**0.941**	**0.547**	**0.994**	**0.865**	**0.987**	**0.965**	**0.973**
**p-value**	**0.002**	**0.204**	**0.001**	**0.012**	**0.001**	**0.001**	**0.001**

Pearson correlation coefficient, r - Conditioned Media

vs.	U6	miR-16	miR-103	miR-106
**miR-16**	0.804	-	-	-
**p-value**	0.054	-	-	-
**miR-103**	0.769	0.965	-	-
**p-value**	0.074	0.002	-	-
**miR-106**	0.678	0.861	0.948	-
**p-value**	0.138	0.028	0.004	-

BestKeeper vs.	U6	miR-16	miR-103	miR-106
**coeff. of corr. [r]**	**0.804**	**0.957**	**0.993**	**0.965**
**p-value**	**0.054**	**0.003**	**0.001**	**0.002**
