# Supplementary material for: Selecting Normalizers for MicroRNA RT-qPCR Expression Analysis in Murine Preimplantation Embryos and the Associated Conditioned Culture Media
Source: J Dev Biol. 2023 Apr 4;11(2):17. doi: 10.3390/jdb11020017 (PMC10123758; doi:10.3390/jdb11020017)
Supplement: Supplementary file 1 [file jdb-11-00017-s001.zip › Table S3.pdf]

Table S3: Regression output describing individual candidate correlation with the BestKeeper index (BK) according to the BestKeeper tool for whole embryo lysates and conditioned media sample sets.

Regression Analysis: HKG vs. BestKeeper - Whole Embryo Lysates

	U6 vs. BK	let-7a vs. BK	miR-16 vs. BK	miR-26a vs. BK	miR-103 vs. BK	miR-106 vs. BK	miR-191 vs. BK
**coeff. of corr. [r]**	0.94	0.55	0.99	0.87	0.99	0.97	0.97
**coeff. of det. [r^2]**	0.89	0.30	0.99	0.75	0.97	0.93	0.95
**intercept [CP]**	-18.92	24.08	1.02	13.62	-11.39	-2.79	-2.05
**slope [CP]**	1.47	0.32	0.91	0.66	1.35	1.11	1.10
**SE [CP]**	±1.315	±1.206	±0.249	±0.948	±0.548	±0.747	±0.644
**p-value**	0.002	0.204	0.001	0.012	0.001	0.001	0.001
**Power of HKG [x-fold]**	2.77	1.25	1.88	1.58	2.55	2.15	2.14

Regression Analysis: HKG vs. BestKeeper - Conditioned Media

	U6 vs. BK	miR-16 vs. BK	miR-103 vs. BK	miR-106 vs. BK
**coeff. of corr. [r]**	0.80	0.96	0.99	0.97
**coeff. of det. [r^2]**	0.65	0.92	0.99	0.93
**intercept [CP]**	3.17	1.43	-1.81	-1.30
**slope [CP]**	0.54	0.90	1.36	1.01
**SE [CP]**	±0.623	±0.427	±0.253	±0.427
**p-value**	0.054	0.003	0.001	0.002
**Power of HKG [x-fold]**	1.45	1.87	2.57	2.01
